# Multiple lung nodules with the halo sign due to syphilis

**DOI:** 10.1590/0037-8682-0246-2023

**Published:** 2023-07-24

**Authors:** Diogo Goulart Corrêa, Rodrigo Paulino, Roberto Mogami

**Affiliations:** 1 Universidade do Estado do Rio de Janeiro, Departamento de Medicina Interna, Disciplina de Radiologia, Rio de Janeiro, RJ, Brasil. Universidade do Estado do Rio de Janeiro Departamento de Medicina Interna Disciplina de Radiologia Rio de Janeiro RJ Brasil; 2 Clínica de Diagnóstico por Imagem/DASA, Departamento de Radiologia, Rio de Janeiro, RJ, Brasil. Clínica de Diagnóstico por Imagem/DASA Departamento de Radiologia Rio de Janeiro RJ Brasil

**FIGURE 1:**
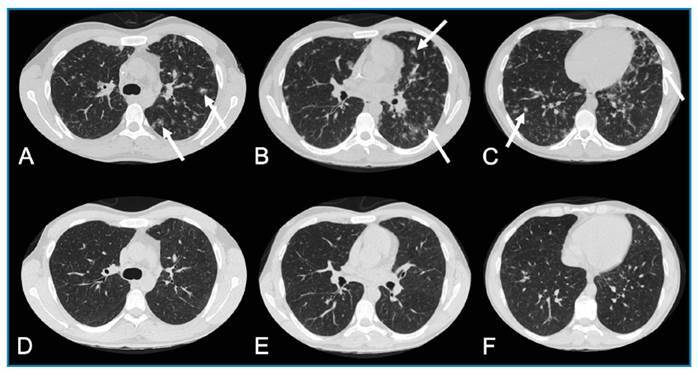
Pulmonary syphilis in a 26-year-old man. **(A-C)** An axial chest CT scan showing multiple nodules in both lungs, some with peripheral ground-glass halos, constituting the halo sign (arrows). **(D-F)** A chest CT scan performed 2 weeks after receiving penicillin G showing the resolution of all lesions.

A 26-year-old man presented with fever, a maculopapular rash on the chest and face, oral and genital ulcers, and cough for 2 weeks. Serological testing revealed positivity for human immunodeficiency virus, with a viral load of 18,218 copies/mL and a CD4 T lymphocyte count of 880 cells/mm^3^. The Venereal Disease Research Laboratory test revealed serum positivity (1:512), and the fluorescent treponemal antibody absorption test was reactive. Chest computed tomography (CT) revealed multiple nodules in both lungs, some with peripheral ground-glass halos. The patient was treated with penicillin G. A chest CT performed 2 weeks after treatment showed resolution of all lesions (Figure 1).

Pulmonary involvement may occur in the secondary stage of syphilis due to the hematogenous spread of *Treponema pallidum* or in the tertiary stage with gummas and/or fibrotic lesions^1^. Our patient met the diagnostic criteria of pulmonary syphilis, which include typical findings of syphilis in other organs, positive serological findings, imaging abnormalities that cannot be explained by other conditions, and response to syphilis treatment^2^. However, pulmonary syphilis can occur without the typical cutaneous lesions^3^. The imaging findings of pulmonary syphilis include multiple nodules^2^ and/or masses^3^ simulating primary lung neoplasia or metastases, and abscesses^1^ with or without mediastinal lymph node enlargement. ^18^F fluorodeoxyglucose (18F-FDG) positron emission tomography-CT may show intense 18F-FDG uptake by the lesions^2^. Although rarely reported, pulmonary syphilis may be under-recognized since chest imaging is not routinely performed. Therefore, syphilis should be included in the differential diagnosis of patients with respiratory symptoms and unexplained lung nodules or masses^3^.
